# Analysis of an RNA-seq Strand-Specific Library from an East Timorese Cucumber Sample Reveals a Complete *Cucurbit aphid-borne yellows virus* Genome

**DOI:** 10.1128/genomeA.00320-17

**Published:** 2017-05-11

**Authors:** Solomon Maina, Owain R. Edwards, Luis de Almeida, Abel Ximenes, Roger A. C. Jones

**Affiliations:** a School of Agriculture and Environment, Faculty of Science, University of Western Australia, Crawley, Western Australia, Australia; b Institute of Agriculture, Faculty of Science, University of Western Australia, Crawley, Western Australia, Australia; c Cooperative Research Centre for Plant Biosecurity, Canberra, Australian Capital Territory, Australia; d CSIRO Land and Water, Floreat Park, Western Australia, Australia; e Seeds of Life Project, Ministry of Agriculture, Forestry and Fisheries, Dili, East Timor; f DNQB-Plant Quarantine, International Airport Nicolau Lobato Comoro, Dili, East Timor; g Department of Agriculture and Food Western Australia, South Perth, Western Australia, Australia

## Abstract

Analysis of an RNA-seq library from cucumber leaf RNA extracted from a fast technology for analysis of nucleic acids (FTA) card revealed the first complete genome of *Cucurbit aphid-borne yellows virus* (CABYV) from East Timor. We compare it with 35 complete CABYV genomes from other world regions. It most resembled the genome of the South Korean isolate HD118.

## GENOME ANNOUNCEMENT

As part of a biosecurity project to examine possible genetic connectivity between viruses infecting crops in northern Australia and nearby Southeast Asian countries, virus genomes from East Timorese and Australian plant samples were compared (1–8). During July 2015, 15 and 22 leaf samples were collected from cucurbit plants with virus-like symptoms in East Timor and northwest Australia, respectively, and subjected to next-generation sequencing. The 15 East Timorese samples were blotted onto fast technology for analysis of nucleic acids (FTA) cards before dispatch to Australia. A complete genome of *Cucurbit aphid-borne yellows virus* (CABYV) was obtained from cucumber (*Cucumis sativus*) sample TM50 from Aileu in East Timor. CABYV was described in 1992 as causing yellow leaf symptoms in cucurbit plants in France (9) and belongs to the genus *Polerovirus* within the family *Luteoviridae* (10). CABYV is a phloem-limited virus transmitted persistently by aphid vectors, including *Aphis gossypii* and *Myzus persicae* (9). It consists of a single-stranded positive-sense RNA molecule with a length of 5.7 kb (10). In sample TM50, analysis of nonpolyadenylated transcripts derived from RNA-seq strand-specific libraries (1–8, 11–14) prepared from RNA extracted from FTA cards (1–8, 15, 16) detected CABYV (designated isolate CABYV AL50).

Total RNA was extracted from FTA card disks using a ZR Plant RNA MiniPrepTM kit (Zymo Research) and treated with RNase-free DNase (Invitrogen). Extracts were subjected to library preparation using a Ribo-Zero plant kit (catalog no. RS-122-2401, Illumina) with the amount of Agencourt AMPure XP beads (Beckman Coulter, Inc.) increased to 20% in every clean-up and no RNA fragmentation step. Libraries were sequenced using a MiSeq platform with a 2 × 251 version 2 kit (Illumina) and a 1% PhiX version 3 spike control. Reads were assembled and genomes annotated using CLC Genomics Workbench version 6.5 (CLC bio) and Geneious version 8.1.7 (Biomatters) (1–8, 17, 18).

CABYV isolate AL50 yielded 4,670,138 reads and, after trimming, 4,058,029 remained. Although AL50’s RNA integrity number (RIN) was only 2, this high number of reads was still obtained. *De novo* assembly generated 229 contigs and 130,416 reads mapped to the contig of interest with 31,129× coverage. The genome obtained had 5,677 nucleotides (nt) and 6 open reading frames (ORFs) organized into 2 clusters, as with other poleroviruses (19). A BLAST-based search (20), revealed that AL50 most resembled the South Korean isolate HD118 (KR231951) with 88.1% nt identity. When AL50’s P3 gene sequence was compared with five CABYV-like P3 sequences from Tasmania, Australia available in GenBank, it most resembled isolate 18 (HQ543088) with 81.2% nt identity. The extent of P3 gene sequence divergence between AL50 and Tasmanian isolate 18 was too great to indicate any genetic connectivity. Further sequencing of CABYV in Tasmania and mainland Australia is required to obtain complete genomes and compare them with genomes from neighboring Southeast Asian countries.

### Accession number(s).

The GenBank accession number is KY617826.
